# External trigeminal nerve stimulation for neuropsychiatric disorders: mechanisms, efficacy, and future directions

**DOI:** 10.3389/fneur.2025.1737506

**Published:** 2026-01-23

**Authors:** Fuqiang Wang, Yaojiang Li, Yunhong Deng, Xiaodi Li, Chunmei Zhao, Liangxiang Zhang, Mengliang Ma, Bianhong He, Xiao Lv, Lixia Deng

**Affiliations:** 1 Department of Rehabilitation Medicine, Guangdong Sanjiu Brain Hospital, Guangzhou, Guangdong, China; 2 Department of Rehabilitation Therapy, Guangdong Sanjiu Brain Hospital, Guangzhou, Guangdong, China; 3 College of Acupuncture and Rehabilitation, Guangzhou University of Chinese Medicine, Guangzhou, Guangdong, China

**Keywords:** brain networks, eTNS, external trigeminal nerve stimulation, neuromodulation, neuropsychiatric disorders, trigeminal pathway

## Abstract

External trigeminal nerve stimulation (eTNS) is an emerging noninvasive neuromodulation technique that modulates brain network activity implicated in multiple neuropsychiatric disorders through the application of low-intensity electrical currents to the frontal branches of the trigeminal nerve. Based on a search of major biomedical databases, we narratively synthesize clinical and mechanistic studies of eTNS across neurological and psychiatric indications. Current clinical and neurophysiological studies suggest clinically meaningful benefits of eTNS in attention-deficit/hyperactivity disorder (ADHD) and migraine, show promising adjunctive efficacy in epilepsy, and may offer emerging therapeutic potential for conditions such as depression, anxiety, and disorders of consciousness. Although eTNS generally demonstrates a favorable safety profile, existing studies are constrained by small sample sizes, short follow-up periods, and considerable heterogeneity in stimulation parameters, and standardized clinical protocols are still lacking. Future research should prioritize multicenter randomized controlled trials, parameter optimization, and biomarker-guided personalized modulation. By integrating neurophysiological, neuroimaging, and behavioral metrics, such efforts may advance eTNS toward a precisely controlled, closed-loop neuromodulation system. This narrative review provides a concept-driven synthesis of the anatomical foundations, putative mechanisms, and clinical evidence for eTNS and outlines key directions for standardization and translational research, thereby providing a reference framework for the clinical implementation of eTNS in neuropsychiatric disorders.

## 1 Introduction

Neuropsychiatric disorders are a complex group of conditions caused by abnormal brain function and are often characterized by cognitive, emotional, and behavioral impairments. Common types include attention-deficit/hyperactivity disorder (ADHD), epilepsy, and depression (1). The Global Burden of Disease study has reported that neurological disorders are now the leading cause of global disease burden, affecting approximately 3.4 billion individuals—around 43% of the world’s population (2). At present, pharmacotherapy remains the mainstay of treatment. However, adverse effects, poor patient adherence, and suboptimal therapeutic responses substantially limit clinical outcomes (3). Therefore, developing safe and effective non-pharmacological interventions has become an urgent priority.

External trigeminal nerve stimulation (eTNS) is an emerging noninvasive neuromodulation technique that applies low–intensity electrical stimulation to the supraorbital branch of the trigeminal nerve through surface electrodes (4–7), thereby modulating neural circuits implicated in diverse neuropsychiatric disorders. The technique was developed in the early 2000s and received FDA approval in 2019 for the treatment of children aged 7–12 years with ADHD, marking its initial clinical recognition. Compared with other neuromodulation modalities, eTNS offers several advantages: it provides targeted precision, allowing more accurate modulation through specific nerve branches (6–8). In addition, the device is safe and portable, making it suitable for home-based use (5, 9, 10). Moreover, eTNS exerts central–peripheral synergistic effects through the trigeminal nerve pathway, thereby achieving multisystem modulation (8, 11–13). These characteristics uniquely position eTNS within the field of neuromodulation, providing a promising treatment option for patients with treatment-resistant neuropsychiatric disorders.

Although eTNS has demonstrated therapeutic potential for neuropsychiatric disorders, several challenges and controversies persist in its clinical application. Treatment responses vary considerably among individuals, and reliable predictive biomarkers remain lacking (7, 13). Currently, no standardized protocol exists for defining optimal stimulation parameters–including intensity, frequency, duration, and treatment cycles–and these parameters differ markedly across studies, thereby affecting the comparability of results and hindering clinical translation (7, 14, 15).

At present, evidence regarding the long-term efficacy and safety of eTNS remains limited (7). Compared with other neuromodulation modalities, the advantages of eTNS still require validation in large-scale, multicenter randomized controlled trials (7, 9, 16). Moreover, the mechanism of action underlying eTNS has not been fully elucidated, thereby constraining its broader clinical application and further development.

This narrative review aims to provide an integrated overview of recent advances, clinical applications, and future directions for eTNS in the management of neuropsychiatric disorders. We summarize the neuroanatomical basis of eTNS, discuss four interrelated mechanistic hypotheses, and integrate representative clinical evidence on its applications across major neuropsychiatric disorders. In light of the limitations of the existing evidence, we outline future research directions centered on mechanistic exploration, parameter optimization, and individualized therapeutic strategies. This synthesis is intended to link mechanistic insights with clinically relevant findings and to provide a conceptual framework to guide the precise clinical application and future research design.

## 2 Literature search and narrative synthesis approach

This article is a narrative review. We conducted a literature search in PubMed, Web of Science, and Embase from database inception to September 13, 2025. The search terms included: “external trigeminal nerve stimulation,” “trigeminal nerve stimulation,” “supraorbital nerve stimulation,” “supraorbital transcutaneous nerve stimulation,” and “eTNS,” combined with relevant subject headings (e.g., “Trigeminal Nerve” combined with “Transcutaneous Electric Nerve Stimulation”) and device names used in eTNS studies (e.g., “Cefaly”). The search was limited to English-language publications. The full electronic search strategy for each database is provided in Supplementary file 1.

We included human clinical studies reporting clinical outcomes of eTNS in neuropsychiatric patients, as well as mechanistic studies related to eTNS (including both human and animal studies). Exclusion criteria included: invasive trigeminal stimulation, studies not related to eTNS or trigeminal stimulation in a neuropsychiatric or mechanistic context, and conference abstracts.

Because the aim was to integrate key mechanistic hypotheses with representative clinical findings, we did not perform PRISMA-ScR–style exhaustive screening, formal risk-of-bias assessment, or meta-analysis, but instead summarized the evidence qualitatively.

## 3 Anatomy and functional characteristics of the trigeminal nerve

The trigeminal nerve, the fifth cranial nerve, is among the largest and most complex of all cranial nerves. It plays a crucial role in sensory transmission of the head and face and in the control of masticatory movements (17, 18). Its unique anatomical structure and functional network serve as the theoretical foundation for eTNS therapy.

The trigeminal nerve is a typical mixed nerve formed by the junction of a large sensory root and a smaller motor root at the ventrolateral aspect of the pons (17, 18). The sensory root originates from the trigeminal ganglion located in the middle cranial fossa and is primarily responsible for transmitting sensory information from most regions of the head and face (17, 18). The motor root arises from the trigeminal motor nucleus in the pons and primarily controls the motor function of the masticatory muscles (17, 19). The anatomical separation of the sensory and motor roots constitutes the morphological basis enabling the trigeminal nerve to perform both sensory afferent and motor efferent functions (17, 20). Yousry et al. precisely mapped the separation between the sensory and motor roots of the trigeminal nerve and their spatial relationship with adjacent blood vessels using high-resolution magnetic resonance imaging. This finding provides important imaging evidence for a deeper understanding of the complex anatomy of the trigeminal nerve (20).

The trigeminal nerve divides into three major branches arising from the trigeminal ganglion: the ophthalmic nerve (V1), the maxillary nerve (V2), and the mandibular nerve (V3). These branches show distinct differences in both their anatomical distribution and functional roles (17, 21). Among them, the ophthalmic nerve (V1) is a purely sensory branch that innervates the forehead, anterior scalp, upper eyelid, eyeball, and anterior nasal cavity (17, 21). Due to its superficial trajectory in the supraorbital region, abundant sensory terminals, and thin subcutaneous tissue, it has become the most frequently selected stimulation target for eTNS (6, 16, 22, 23). The maxillary nerve (V2) is also a purely sensory branch that conveys sensory information from the midface, upper teeth, maxillary sinus, nasal cavity, and upper lip. Owing to its relatively superficial course in the cheek region, it also shows potential as a target for therapeutic stimulation (21, 24). The mandibular nerve (V3), a mixed branch, contains sensory fibers that supply the lower lip, lower teeth, mandible, and submandibular region, while its motor fibers innervate the muscles of mastication (25, 26). The precise anatomical distribution of the trigeminal nerve not only determines its functional characteristics under physiological and pathological conditions but also forms the anatomical basis for identifying optimal stimulation targets for eTNS.

The trigeminal nerve comprises various types of functional afferent fibers. Large, myelinated Aβ fibers primarily mediate tactile and pressure sensations, whereas medium-diameter, thinly myelinated Aδ fibers are responsible for fast, sharp pain and temperature perception. Small-diameter unmyelinated C fibers predominantly transmit dull and chronic pain signals (27–30). This diversity in nerve fiber types offers multiple potential functional targets for eTNS-based interventions (12). Adjusting stimulation parameters—such as pulse frequency, pulse width, and intensity—modulates the types of fibers recruited and the resulting peripheral and central responses, thereby achieving more targeted therapeutic effects. However, the selective activation of a single fiber type *in vivo* remains technically and physiologically challenging (31–34).

Regarding central conduction pathways, the trigeminal system displays complex, multilevel conduction properties. Sensory information is transmitted via primary afferent fibers to the trigeminal ganglion, where it undergoes preliminary integration and processing within the pontine sensory nucleus and spinal trigeminal nucleus (21, 35, 36). After relaying through the thalamus, the signals ascend via the thalamo-cortical pathway to the primary sensory cortex, thereby completing the trigeminal sensory conduction pathway (21, 36, 37). Functional magnetic resonance imaging (fMRI) studies have demonstrated extensive functional connectivity between the trigeminal system and higher-order brain regions, including the limbic system and prefrontal cortex (PFC) (38–40). This complex neural network not only mediates basic sensory transmission but also contributes to emotional regulation and cognitive processing (40). Alterations in this functional connectivity are closely linked to various neuropsychiatric disorders, providing a theoretical rationale for applying eTNS in their treatment (5, 40, 41).

Given its anatomical and functional features, the trigeminal nerve offers several advantages as a target for neuromodulation. Its superficial branches allow percutaneous stimulation, its diverse sensory fiber composition enables differentiated therapeutic effects, and its broad central functional connectivity provides a strong theoretical basis for applications across neuropsychiatric disorders. Together, these anatomical and functional attributes define the clinical relevance of eTNS in the treatment of neuropsychiatric disorders.

## 4 Hypotheses on the mechanism of action of eTNS

**Figure 1:**
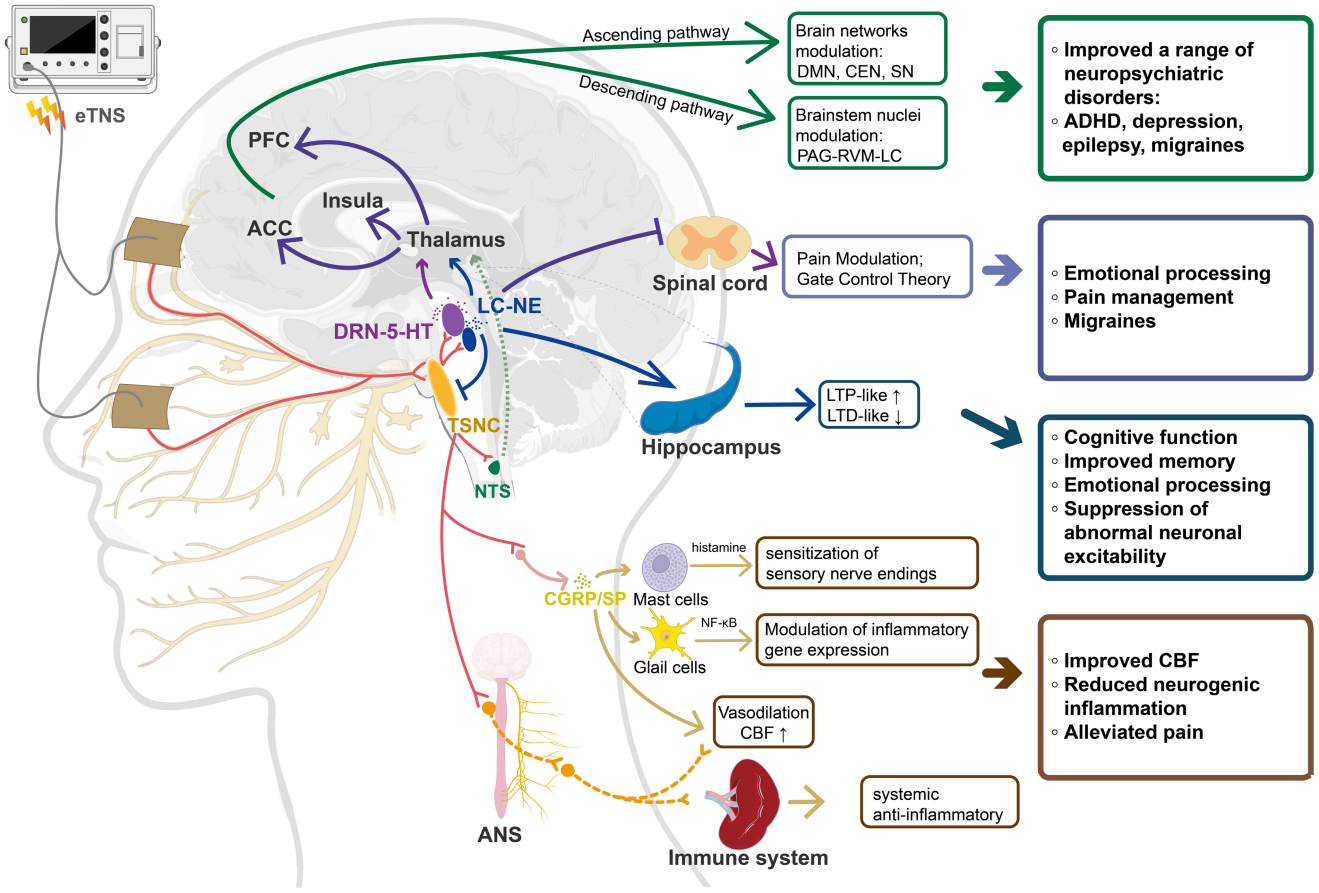
Schematic illustration of the proposed multi-level mechanisms underlying eTNS. ETNS may exert its therapeutic effects via four interrelated mechanisms: (1) Modulation of diffuse neurotransmitter systems (LC–NE, DRN–5-HT); (2) regulation of synaptic and network plasticity; (3) neurovascular–immune interactions involving neuropeptides and cerebral perfusion; and (4) large-scale network reorganization mediated by bottom-up (TG–TSNC–Thalamus–Cortex) and top-down (PFC/ACC → PAG–RVM–LC) pathways. eTNS, external trigeminal nerve stimulation; LC–NE, locus coeruleus–norepinephrine system; DRN–5-HT, dorsal raphe nucleus–serotonin system; TG, trigeminal ganglion; NTS, nucleus of the solitary tract; TSNC, trigeminal sensory nuclear complex; PFC, prefrontal cortex; ACC, anterior cingulate cortex; PAG–RVM–LC, periaqueductal gray–rostral ventromedial medulla–locus coeruleus; DMN, default mode network; CEN, central executive network; SN, salience network; LTP/LTD, long-term potentiation/depression; CGRP, calcitonin gene–related peptide; SP, substance P; CBF, cerebral blood flow; ANS, autonomic nervous system.

The biological effects of eTNS are mediated through multiple pathways, following a complex trajectory from peripheral trigeminal afferents to brainstem modulation, thalamic gating, cortical–limbic integration, and descending control, thus achieving multilevel signal transmission and regulation. Based on converging evidence from neuroimaging, physiological markers, and animal studies, the mechanism of eTNS can be conceptualized as comprising four complementary and interconnected components. The neurotransmitter regulation hypothesis highlights the critical involvement of key brainstem nuclei—namely, the locus coeruleus–norepinephrine (LC–NE) system and the dorsal raphe nucleus–serotonin (DRN–5-HT) system—in rapid neuromodulatory processes. Building on this foundation, eTNS induces neuroplastic adaptations at both the synaptic and network levels by bidirectionally modulating long-term potentiation (LTP) and long-term depression (LTD), thereby attenuating abnormal neural excitation. Secondly, the hypothesis concerning the interaction among neural signaling, cerebral blood flow, and immune responses posits that eTNS may modulate inflammatory and pain pathways via neuropeptide release, alterations in cerebral hemodynamics, and regulation of the autonomic nervous system, thus exerting broader physiological and pathophysiological effects. In addition, eTNS may produce synergistic modulation through bottom-up peripheral afferents and top-down cortical control, thereby facilitating dynamic coordination among large-scale brain networks, including the default mode network (DMN), central executive network (CEN), and salience network (SN). These mechanisms are schematically summarized in Figure 1.

### 4.1 Neurotransmitter regulation hypothesis

eTNS activates the cutaneous branches of the trigeminal nerve and transmits signals through the trigeminal ganglion and trigeminal sensory nuclei to key regions in the brainstem and thalamus (7, 12), thereby modulating diffuse neurotransmitter networks, including the LC–NE and DRN–5-HT systems (7, 12, 42). These neurotransmitter systems play an essential role in maintaining cortical excitatory–inhibitory balance and network plasticity (43–45), thereby providing the physiological basis for the multi-target effects of eTNS on attention, pain perception, and emotion (5, 16, 46, 47).

At the level of the LC–NE axis, the trigeminal nerve may modulate norepinephrine release via its anatomical and functional connections to the brainstem LC–NE system (11). The LC–NE system projects widely to both cortical and subcortical regions, where it contributes to the regulation of arousal, attention, learning and memory, emotion, as well as pain processing (48, 49). Activation of this system by eTNS may enhance norepinephrine transmission and modulate neural activity within stress- and emotion-related networks, thereby affecting pain sensitivity and emotional responses (11, 42, 48, 50). Concurrently, the LC is involved in pain gating by inhibiting the trigeminal sensory nuclear complex (TSNC) and modulating spinal and medullary nociceptive pathways (42). This mechanism is consistent with clinical evidence supporting the efficacy of eTNS in migraine prevention and acute therapy (9, 16, 46).

At the level of the DRN–5-HT axis, the DRN–5-HT system exerts bidirectional effects on pain–emotion processing, mediated by distinct receptor subtypes in the anterior cingulate cortex (ACC) and the insula (51, 52). In the dorsal horn of the spinal cord, its influence on nociceptive transmission can manifest as either inhibition or facilitation, depending on the receptor subtype and the physiological or pathological state (53). This bidirectional mechanism provides a neurobiological basis for the dual modulatory effects of eTNS on mood and pain (12).

Basic experimental evidence further supports these proposed mechanisms. *In vivo* spinal cord perfusion experiments demonstrated that stimulation of the infraorbital branch of the trigeminal nerve in cats induced the release of NE and serotonin 5-HT into the spinal cord perfusate. This effect was abolished by cervical cold block, indicating that trigeminal sensory input activates descending monoaminergic pathways within the brainstem (54). In rodent models, trigeminal-related nociceptive stimulation (e.g., intracisternal capsaicin injection) induces a rapid increase in expression of the early gene product c-Fos within the locus coeruleus, dorsal raphe nuclei, and the medullary nucleus of the solitary tract/parabrachial nucleus, indicating activation of these brainstem regions (55). Mechanical stimulation of the dental pulp or incisor likewise induced c-Fos expression in the locus coeruleus and dorsal raphe nuclei, suggesting anatomical and functional connectivity between trigeminal afferent inputs and these brainstem nuclei (56). Electrophysiological recordings have shown that pulp stimulation activates noradrenergic neurons within the locus coeruleus, accompanied by inhibitory feedback from the locus coeruleus to the spinal trigeminal nucleus (57). Similarly, other noxious stimuli selectively activate locus coeruleus neurons projecting to the somatosensory thalamus, indicating a functional link between pain processing and arousal regulation (58). In a rat model of hemorrhagic shock, trigeminal nerve stimulation elevated plasma NE levels, improved cerebral perfusion, and increased short-term survival, suggesting that eTNS enhances regulation of autonomic output via brainstem monoaminergic systems (59).

Based on current evidence, eTNS may exert its therapeutic effects through the integration of the following neural pathways: Trigeminal afferent signals are first integrated within the trigeminal sensory nucleus and spinal trigeminal tract nucleus, subsequently activating the LC–NE and DRN–5-HT systems. This pathway projects upward through the thalamus to engage cortical control networks, including the prefrontal–cingulate circuitry, thereby enhancing arousal, attention, and executive function. It also projects downward to the spinal dorsal horn and the spinal trigeminal nucleus, where it bidirectionally regulates pain and emotional responses through distinct receptor subtypes and state-dependent mechanisms. Together, these pathways provide a mechanistic basis for the cross-symptomatic therapeutic effects of eTNS across multiple neuropsychiatric disorders.

### 4.2 Hypothesis of neural plasticity regulation

eTNS may alleviate clinical symptoms of neuropsychiatric disorders by modulating neural circuit plasticity. Neural plasticity refers to adaptive alterations in the structure and function of the nervous system, including reversible modifications in synaptic transmission efficiency, neuronal activity patterns, and large-scale network connectivity. eTNS is believed to influence these processes and exert therapeutic effects across various disorders, particularly by modulating synaptic transmission, facilitating network remodeling, and strengthening inhibitory circuits.

eTNS dynamically regulates synaptic transmission efficiency through activity-dependent mechanisms of synaptic plasticity, thereby modifying information transfer between neurons (60). Animal studies have shown that trigeminal nerve stimulation enhances neuronal firing in specific brain regions and may influence *β*-adrenergic receptor–mediated synaptic plasticity through activation of the LC–NE system (61). LC–NE signaling can regulate long-term synaptic plasticity in the hippocampus and lower the induction threshold of LTP (62, 63). Additionally, direct LC activation can induce LTP formation in the dentate gyrus (64). Therefore, it is speculated that eTNS may regulate hippocampal synaptic plasticity through the LC–NE–*β* receptor pathway, thereby promoting the remodeling of neural networks associated with learning and memory. Although this hypothesis provides theoretical support for the application of eTNS in cognitive function and memory recovery, there is still a lack of experimental evidence that eTNS directly induces hippocampal LTP, and further experiments are needed to verify the specific mechanism.

In addition to modulating synaptic plasticity and network excitability, eTNS may enhance inhibitory circuit function by normalizing aberrant neural activity (60, 65). This mechanism may hold therapeutic potential for disorders characterized by hyperexcitability of neural networks, such as epilepsy and migraine. Studies have shown that eTNS modulates downstream neural circuits (such as the LC–NE system) through regulation of trigeminal nerve activity (11, 66), altering neuronal firing patterns and synaptic efficacy, thereby reinforcing inhibitory networks and enhancing overall neural function (60, 65, 67).

In terms of manifestations of plasticity, the typical forms of synaptic plasticity are LTP and LTD, which represent increases and decreases in synaptic transmission efficiency, respectively (68). eTNS can elicit LTP-like or LTD-like synaptic responses, thereby modulating neural circuit dynamics under varying conditions (60, 69–71). In human studies, trigeminal nerve stimulation has been shown to enhance blink reflex pathway efficacy, an effect interpreted as LTP-like plasticity (71). Low-frequency stimulation protocols have demonstrated LTD-like effects across multiple studies, including prolonged suppression of the blink reflex (60, 69–71) and modulation of sensory and nociceptive processing (70, 72). Furthermore, animal studies have demonstrated that trigeminal nerve stimulation enhances hippocampal plasticity and upregulates related functional markers (e.g., c-Fos expression and cell proliferation), thereby providing physiological evidence supporting the use of eTNS for neuropsychiatric disorders associated with network dysfunction (73, 74).

In conclusion, eTNS enhances synaptic transmission and information-processing capacity through LTP-like effects and inhibits abnormal neuronal excitation through LTD-like effects. This bidirectional regulatory mechanism may constitute the neurobiological basis underlying the therapeutic effects of eTNS across various neuropsychiatric disorders.

### 4.3 Neurovascular–immune regulation hypothesis

The therapeutic effects of eTNS are closely associated with neurovascular–immune regulatory mechanisms. It exerts its therapeutic effects by coordinating the regulation of cerebral vasodilation, brain metabolism, and the autonomic nervous system (12, 75). This mechanistic framework not only involves the regulation of cerebral hemodynamics and vasoactive substances but also includes the control of neurogenic inflammation and immune responses.

At the neurovascular regulatory level, eTNS may modulate cerebral blood flow through two primary pathways. First, activation of the trigeminal–parasympathetic reflex pathway directly increases cerebral blood flow (CBF) and enhances local cerebral perfusion (75, 76); second, it modulates the release of vasoactive neuropeptides, including calcitonin gene-related peptide (CGRP) and substance P (SP), thereby promoting vasodilation (12, 76–78). These neuropeptides act on nerve endings and vascular walls, participating in the regulation of vascular tone and contributing to neurogenic inflammatory processes (12, 77). CGRP and SP may synergistically regulate local blood flow and induce neurogenic inflammation, thereby strengthening the bidirectional interaction between blood vessels and sensory nerves (12, 77–79). The release of these peptides also serves as a critical link in subsequent neuroimmune regulation.

In the context of neuroimmune regulation, eTNS activates the trigeminal nervous system, facilitating the release of neuropeptides such as CGRP at nerve endings and within the microenvironment of the trigeminal ganglion, thereby modulating the neuroimmune axis. CGRP not only induces meningeal vasodilation but also activates mast cells to release pro-inflammatory mediators, enhances sensory-nerve sensitization, and increases vascular permeability, thereby amplifying the inflammatory response (80). Simultaneously, CGRP activates the nuclear factor-κB (NF-κB) signaling pathway in glial cells of the trigeminal ganglion, thereby regulating the expression of inflammation-related genes (81). This process further increases nociceptor sensitivity through a positive feedback mechanism, thereby sustaining pain perception (80, 82). Clinical studies have also demonstrated that CGRP levels in peripheral blood—particularly in the external jugular vein—are significantly elevated during migraine attacks, further supporting the pivotal role of neurovascular–immune interactions in the pathophysiology of migraine (83–85).

Additionally, eTNS may indirectly influence immune responses by regulating autonomic nervous system (ANS) function. By modulating the balance between the sympathetic and parasympathetic nervous systems, eTNS may alter blood perfusion and immune cell activity in organs such as the spleen and lymph nodes, thereby exerting systemic anti-inflammatory effects (86, 87).

In summary, the neurovascular–immune regulation hypothesis offers a multilayered framework for elucidating the therapeutic mechanisms of eTNS. The synergistic regulation of cerebral blood flow, neuropeptide release, neurogenic inflammation, and autonomic nervous activity collectively underscores the therapeutic potential of eTNS across diverse neuropsychiatric disorders.

### 4.4 The hypothesis of multi-level neural circuit integration

The hypothesis of multi-level neural circuit integration proposes that eTNS modulates neural functions at multiple levels through a bidirectional regulatory mechanism involving bottom-up afferent inputs and top-down cortical–brainstem pathways, thereby providing a biological basis for its broad applicability across neurological and psychiatric disorders.

eTNS activates the trigeminal nerve via frontal electrodes. The electrical signal is conveyed via the trigeminal ganglion to the TSNC and subsequently projected to multiple brainstem regulatory nuclei, including the LC–NE system, the DRN–5-HT system, and the nucleus of the solitary tract (NTS) (11, 12, 66, 73, 88). Human neurophysiological studies indicate that eTNS exerts a stronger modulatory effect on brainstem excitability than on cortical activity, reflecting its predominantly bottom-up driving characteristics (89). Subsequently, the trigeminal afferent inputs ascend to the cerebral cortex and limbic structures (such as the amygdala and hippocampus) via thalamic gating mechanisms involving thalamocortical relay nuclei and the thalamic reticular nucleus, thereby influencing large-scale brain networks such as the DMN, CEN, and SN (38, 41, 90–93). These networks play pivotal roles in emotional regulation, cognitive control, and pain processing (94–97). The thalamus not only transmits and integrates sensory information but also modulates attention and emotional states through its reciprocal interactions with the cortex.

In addition to bottom-up pathways, eTNS also regulates neural activity through top-down cortical–brainstem circuits. The PFC, ACC, and insula serve as core regions involved in emotional and cognitive regulation. These cortical regions project to the brainstem through descending pathways, such as the PAG–RVM–LC axis, thereby modulating pain processing and emotional reactivity (52, 98–101). These regions not only mediate higher-order cognitive evaluation and emotional appraisal but also modulate the activity of brainstem nuclei, which in turn influence sensory afferent processing.

Neuroimaging evidence provides empirical support for the aforementioned multi-level regulatory mechanisms. Following eTNS treatment, increased glucose metabolism and enhanced functional activation were observed in the orbitofrontal cortex (OFC) and ACC, which correlated with clinical symptom improvement (92, 93). Under noxious stimulation, task-based fMRI has demonstrated increased activation in the anterior hypothalamus and the ventral posteromedial (VPM) nucleus of the thalamus (38, 102, 103). Collectively, these findings support the existence of a hierarchical transmission pathway linking the brainstem, thalamus, cortex, and limbic system (38, 104).

In summary, the multi-level neural circuit integration hypothesis posits that eTNS engages bidirectional synergistic mechanisms via ascending brainstem–thalamic inputs and descending prefrontal–anterior cingulate modulation, thereby establishing integrated multi-level circuitry across the brainstem–thalamus–cortex–limbic axis (38, 104, 105). This integrative mechanism enables eTNS to exert broad therapeutic effects across a spectrum of neuropsychiatric disorders, including ADHD, depression, anxiety, epilepsy, and migraine. The capacity of eTNS to evoke diverse therapeutic responses from a single stimulation site underscores the cross-diagnostic applicability of its neural circuit integration mechanism (5, 7, 46, 106, 107).

## 5 Clinical application

eTNS has been investigated in a range of neuropsychiatric disorders, including ADHD, migraine, epilepsy, depression, and others. Key clinical studies and their main characteristics are summarized in Table 1.

**Table 1:** Key clinical studies of eTNS in neuropsychiatric disorders.

Indication	Study	Design	Population	Stimulation protocol	Outcomes	Adverse events
ADHD	McGough, 2019 (5)	Randomized, double-blind, sham-controlled trial	62 children with ADHD	120 Hz, 250 μs, 30 s on / 30 s off; ~8 h/night during sleep for 4 weeks	Active eTNS significantly improved ADHD Rating Scale scores vs. sham, with effects persisting ≥1 week post-stimulation.	Mild (sleep-related symptoms, drowsiness, headache)
Migraine (acute)	Chou, 2019 (46)	Randomized, double-blind, sham-controlled trial	106 adults with migraine	100 Hz, 250 μs, max 16 mA; single 60-min session	Verum eTNS reduced pain intensity significantly more than sham at 1 h.	Mild (transient nausea, painful paresthesia)
	Kuruvilla, 2022 (9)	Randomized, double-blind, sham-controlled (Phase 3)	538 adults with migraine	100 Hz, 250 μs, max 16 mA; single 2 h session	Higher 2-h pain freedom and MBS resolution vs. sham.	Most common was forehead paresthesia/discomfort/burning
Migraine (prevention)	Schoenen, 2013 (16)	Randomized, double-blind, sham-controlled	67 adults with migraine	60 Hz, 250 μs, max 16 mA; 20 min/day for 3 months	Verum significantly reduced monthly migraine days and increased the 50% responder rate vs. sham	No adverse events
DRE	DeGiorgio, 2013 (106)	Randomized, double-blind, active-control trial	50 adults with focal DRE	120 Hz, <250 μs, ≥12 h/day for 18 weeks vs. 2 Hz (2 s on/90 s off), 50 μs, ≥12 h/day for 18 weeks	No significant between-group seizure difference over 18 wks.; mood improved more with 120 Hz	Minor (anxiety, headache, skin irritation)
	Soss, 2015 (110)	Prospective open-label long-term study	35 adults with DRE	120 Hz, 30 s on/30 s off, 250 μs	Median seizure frequency decreased 27–35% at 6 and 12 months; ≥50% responder rate ~37%	No adverse events
	Gil-López, 2020 (47)	Randomized controlled trial	40 adults with focal DRE	120 Hz, <10 mA, 250 μs, 30 s on/30 s off; ≥8 h/day	50% responders at 12 months; ~43.5% seizure-frequency reduction vs. baseline	No adverse events
MDD	Cook, 2013 (111)	Open-label pilot study	11 adults with MDD	120 Hz, 4–6 mA, 250 μs, 30 s on/30 s off; 8 h/night during sleep for 8 weeks	Significant improvement in depressive symptoms and quality of life; 4/11 achieved remission.	No adverse events
	Shiozawa, 2015 (112)	Randomized, sham-controlled Phase II trial	40 adults MDD	120 Hz, 250 μs; 30 min/session; 10 weekday sessions over 2 weeks	Active eTNS produced significantly greater reduction in HDRS-17 scores versus sham	No adverse events
	Generoso, 2019 (107)	Randomized, sham-controlled Phase II trial	24 adults with MDD	120 Hz, 250 μs; 30 min/session; 10 weekday sessions over 2 weeks	Active eTNS showed greater HDRS-17 improvement vs. sham, persisting at 1-month follow-up	Mild local paresthesia; no severe adverse effects
GAD	Trevizol, 2015 (114)	Single-patient case study	1 adult with GAD	120 Hz, 250 μs; 30 min/day, for 10 days	Symptomatic remission with marked anxiety reduction sustained at 1 month; MoCA improved.	No adverse events
SAD	Trevizol, 2016 (115)	Single-patient case study	1 adult with SAD	120 Hz; 250 μs; 30 min/day, for 10 days	Significant reduction in anxiety and depressive symptoms, with 50% improvement in anxiety symptoms after 10 sessions	No adverse events
PTSD	Cook, 2016 (116)	Open-label, pilot study	10 adults with both PTSD	120 Hz, 250 μs, 4–6 mA, ~30 s on/30 s off; 1 h/day, for 4 weeks	Significant reductions in PTSD and depressive symptoms over 8 weeks, with large effect sizes and improved quality of life	Mild (mainly skin irritation or headache); none serious
Insomnia disorder	Um, 2022 (117)	Prospective, single-arm pilot study	13 patients with insomnia	60 Hz, 250 μs, max 16 mA; 20 min/day, for 4 weeks	Significant improvement in PSQI, ISI, and ESS scores after 4 weeks	Mild and transient discomfort or skin sensations
DOC	Fan, 2019 (118)	Single-patient case report	1 adult patient with DOC	40 Hz, 200 μs, 18–20 mA; 30 s on/30 s off; 6 h/day, for 6 weeks	Improvement in consciousness and GCS score after 6 weeks	No adverse events
	Wu, 2022 (119)	Randomized, double-blind, sham-controlled study	63 adults with DOC	140/28 Hz, 8 mA; 40 min/day, for 5 consecutive days	Improvement in consciousness in 43.5% (gamma) and 25% (beta) of patients, with sustained effects at 1-year follow-up.	Mild (discomfort or skin irritation)
	Ma H, 2023 (91)	Randomized, double-blind, sham-controlled trial	60 adults with DOC	40 Hz, 200 μs, 10–15 mA, 30 s on/30 s off; 3 h/day, 5 days/week for 4 weeks	Improved consciousness (CRS-R) compared with sham.	No adverse events

DRE, drug-resistant epilepsy; MDD, major depressive disorder; GAD, generalized anxiety disorder; SAD, social anxiety disorder; PTSD, posttraumatic stress disorder; DOC, disorders of consciousness.

### 5.1 Application of eTNS in ADHD

eTNS has demonstrated considerable therapeutic potential in the treatment of ADHD, potentially modulating the prefrontal network via the “trigeminal nerve–brainstem–thalamic” pathway, regulating the excitatory–inhibitory balance within the cerebral cortex, and thereby improving core symptoms of ADHD, including attention deficits and impaired self-control (5, 7).

In April 2019, the U. S. FDA approved the Monarch eTNS System, the first eTNS device indicated for the treatment of children aged 7–12 years with ADHD, thereby establishing it as the first nonpharmacologic therapy approved for this disorder. This approval was primarily supported by a randomized controlled trial (RCT) conducted by McGough et al., which showed that following a four-week intervention, pediatric participants exhibited significant improvements on the Clinical Global Impression–Improvement Scale and the ADHD Rating Scale. Furthermore, the therapeutic benefits were maintained for at least 1 week after treatment discontinuation (5). However, the relatively small sample size may have limited the statistical power and generalizability of its findings. Additionally, despite the double-blind design, the sensory sensations induced by eTNS could have allowed certain participants and evaluators to differentiate real from sham stimulation, thereby introducing potential unblinding bias. This limitation could undermine the reliability of subjective outcome measures. Subsequent analysis by Loo et al. indicated that children with ADHD exhibiting executive dysfunction were more likely to respond favorably to eTNS, showing modulation of right frontal lobe activity, enhanced executive functioning, and symptom reduction (13). These findings suggest that eTNS may be particularly effective for certain ADHD subtypes.

Although current evidence supports the short-term symptomatic improvement and favorable safety profile of eTNS in pediatric ADHD, large-sample, multicenter, and long-term follow-up studies are still lacking to comprehensively evaluate its clinical efficacy. Furthermore, no studies have directly compared the relative efficacy and safety of eTNS with established first-line interventions.

Therefore, several critical methodological issues warrant attention in future research. Further multicenter RCTs with larger sample sizes are required to strengthen the robustness of the findings; more rigorous blinding procedures should be designed to minimize the risk of unblinding—such as refining sham stimulation protocols or incorporating objective outcome measures; and long-term follow-up studies should be conducted to assess the durability of therapeutic effects and long-term safety.

### 5.2 Application of eTNS in migraine

eTNS exerts its therapeutic effects by stimulating the trigeminal nerve, thereby modulating the trigeminal–cerebrovascular–pain pathway associated with migraine. Brain imaging and electrophysiological studies have demonstrated that eTNS modulates neural activity in regions involved in pain processing, such as the ACC and OFC, and alters cortical responses within the trigeminal sensory pathway. Therefore, it is hypothesized that eTNS alleviates migraine by enhancing descending inhibitory pain pathways and reducing pain-related afferent signaling within the trigeminal system, thereby decreasing overall excitability along the trigeminal–brainstem–cortical pathway (92, 93, 108).

In the acute treatment of migraine, eTNS is supported by a growing body of clinical evidence. A randomized, double-blind, sham-controlled trial demonstrated that a 1 h eTNS session administered during migraine attacks significantly reduced pain intensity, increased pain relief rates, and raised the proportion of patients who achieved complete pain freedom (46). The Phase III TEAM study further confirmed that the eTNS group achieved significantly higher 2 h pain-free and most bothersome symptom relief rates than the sham group (9). *Post hoc* analyses indicated that eTNS consistently alleviated photophobia and nausea; however, its effects on phonophobia and vomiting remain inconclusive and warrant further investigation (109).

In migraine prevention, randomized, sham-controlled trials demonstrated that a daily 20 min eTNS regimen administered for 3 months significantly reduced monthly migraine days, increased the 50% responder rate (i.e., ≥50% reduction in attack frequency in at least half of patients), and reduced the use of acute analgesics (16). PET imaging studies have further shown that long-term eTNS normalizes metabolic activity in brain regions involved in emotional and pain regulation, suggesting sustained effects mediated by neuroplastic mechanisms (92). Therefore, eTNS is particularly suitable for patients experiencing frequent attacks, those intolerant to medications, or those seeking to minimize drug use, and represents a convenient home-based preventive therapy.

Based on current evidence, early initiation of eTNS during the acute phase of migraine is recommended to maximize pain-free and symptom relief rates. During the preventive phase, consistent eTNS sessions should be maintained to reduce attack frequency and dependence on medication. As a non-pharmacological, home-based intervention, eTNS can be safely combined with pharmacotherapy, offering an alternative for migraine patients who are unsuitable for or unwilling to use medication.

### 5.3 Application of eTNS in epilepsy

The application of eTNS in the treatment of epilepsy is grounded in its neuroanatomical basis and network regulatory mechanisms. The sensory afferent pathways of the trigeminal nerve are closely connected to regions such as the brainstem reticular formation, the LC–NE system, and the thalamus. These structures collectively contribute to the regulation of thalamocortical rhythms and the balance between cortical excitation and inhibition, the latter being a key factor in epileptic discharges. Therefore, eTNS may modulate these central networks through peripheral sensory input, thereby increasing seizure thresholds, suppressing abnormal synchronized discharges, and enhancing mood and alertness in individuals with epilepsy (7, 47, 106).

Multiple clinical studies have investigated the therapeutic efficacy of eTNS in patients with drug-resistant epilepsy (DRE). An RCT involving 50 adult patients with DRE compared the therapeutic effects of stimulation at 120 Hz versus 2 Hz over an 18-week treatment period. The results indicated that the proportion of patients achieving > 50% reduction in seizure frequency in the treatment group gradually increased over time (106). In a long-term open-label extension of this trial, Soss et al. reported median reductions in seizure frequency of 27 and 35% at 6 and 12 months relative to baseline, respectively, with an overall ≥50% responder rate of approximately 31% (110). Taken together, the progressive increase in ≥50% responders during the blinded phase and the additional seizure reduction observed in the extension phase support the notion that the therapeutic efficacy of eTNS may accumulate over time. Another single-center RCT enrolled 40 patients with frontal or temporal lobe DRE; after 12 months of follow-up, the 50% responder rate in the eTNS group reached 50%, with a median seizure-frequency reduction of 43.5%, compared with 0% in the control group. Patients with temporal lobe epilepsy demonstrated slightly greater improvement than those with frontal lobe epilepsy (47).

Based on the current evidence, eTNS represents a safe, well-tolerated, and convenient noninvasive adjunctive therapy for adult patients with focal DRE. Patients with temporal lobe epilepsy appear to be more responsive to eTNS, and its therapeutic efficacy shows a cumulative pattern over time.

### 5.4 Application of eTNS in depression

eTNS therapy for depression is supported by a plausible neurobiological basis, though direct and systematic mechanistic evidence is still lacking. The facial branches of the trigeminal nerve project through the TSNC to brainstem regions including the NTS, thereby modulating key neuromodulatory nuclei such as the LC–NE and DRN–5-HT systems. These nuclei are functionally interconnected with the PFC, ACC, insula, and other cortical regions, contributing to the regulation of emotion and cognition. Therefore, stimulation of the trigeminal nerve may modulate an individual’s alertness and processing of external information, thereby reducing negative affect (7).

Early clinical evidence has suggested that eTNS has therapeutic potential in the treatment of depression. An open-label study conducted by Cook et al. included 11 patients with major depressive disorder (MDD). Following 8 weeks of nocturnal eTNS treatment, significant reductions were observed across all clinical scales, and no serious adverse events were reported. This finding provides preliminary support for future controlled studies (111). A subsequent small-sample, double-blind, sham-stimulation-controlled Phase II randomized trial found that after 10 days of eTNS treatment, patients exhibited a significant reduction in Hamilton Depression Rating Scale (HDRS-17) scores compared to baseline, with no serious adverse events reported (112). Another study involving 24 participants with MDD similarly reported significant improvements in depressive symptoms following 10 days of treatment. The between-group differences persisted for at least 1 month after treatment discontinuation, suggesting that short-term eTNS may exert sustained antidepressant effects (107). This persistence may be attributed to the long-lasting modulatory influence of eTNS on brain neural networks. A systematic review and meta-analysis further confirmed that eTNS is associated with significant improvement in depressive symptoms and is generally well tolerated (7).

Although existing evidence supports the antidepressant effects of eTNS, the overall scale and methodological quality of current studies remain limited. Furthermore, direct comparative studies with established first-line treatments, such as selective serotonin reuptake inhibitors (SSRIs) or repetitive transcranial magnetic stimulation (rTMS), are lacking. Therefore, it is premature to regard eTNS as a first-line therapeutic alternative for depression (7). However, evidence regarding its efficacy in specific patient populations remains scarce, and the optimal target population has yet to be determined. Individualized risk–benefit assessment is therefore recommended in clinical practice (7).

### 5.5 Application of eTNS in anxiety disorders

Currently, evidence supporting eTNS for the treatment of anxiety disorders remains at a preliminary, exploratory stage. Despite plausible underlying neurobiological mechanisms, the available evidence remains insufficient to support eTNS as a standard treatment for anxiety disorders. The potential mechanisms may involve the anatomical and functional pathways of the trigeminal nerve. Trigeminal sensory afferent fibers project extensively to brainstem sensory nuclei and, through connections with brainstem monoaminergic networks, may indirectly modulate nodes within forebrain emotion-regulation circuits, such as the PFC and limbic system (12). Additionally, eTNS may indirectly modulate emotional processing and arousal by influencing the LC–NE and DRN–5-HT systems (12, 45, 113).

At present, clinical evidence supporting eTNS for the treatment of anxiety disorders is derived primarily from case reports and small open-label studies, and rigorously designed RCTs remain lacking. In patients with generalized anxiety disorder and social anxiety disorder, short-term eTNS interventions have been associated with reductions in symptom scale scores. However, the reliability of these findings is constrained by very small sample sizes and the lack of appropriate control groups (114, 115). An open-label study investigated the effects of eTNS in patients with MDD comorbid with post-traumatic stress disorder. After 8 weeks of treatment, improvements were observed in clinical symptoms, depressive severity, and quality of life. However, these preliminary findings require confirmation in larger, well-controlled RCTs (116). Systematic reviews and meta-analyses have noted that studies investigating eTNS for psychiatric indications (including anxiety disorders) are limited by small sample sizes, inadequate blinding and control conditions, inconsistent stimulation parameters, short follow-up durations, and substantial heterogeneity in outcome measures, leading to an overall low level of evidence (7).

Based on the currently available evidence, eTNS cannot be recommended as a routine or standard adjunctive therapy for anxiety disorders at present. For treatment-resistant patients with poor responses to medication and psychotherapy, if eTNS is considered for clinical use, they should be fully informed of the limited evidence base and potential placebo effects. During treatment, therapeutic efficacy and adverse events should be closely monitored, and intervention strategies adjusted as appropriate. Future studies should employ rigorously designed RCTs to further evaluate the therapeutic efficacy and safety of eTNS in anxiety disorders.

### 5.6 Other potential applications

In the treatment of sleep disorders, eTNS has shown promising therapeutic potential. A small-sample prospective study reported that a four-week eTNS intervention administered once daily for 20 min significantly improved subjective sleep quality and reduced insomnia severity, whereas no significant changes were detected in polysomnography-derived objective sleep parameters (117). Imaging studies further revealed that after eTNS intervention, resting-state functional connectivity between the LC and the occipital and temporal cortices was significantly decreased. This finding supports the mechanistic hypothesis that eTNS modulates arousal through the noradrenergic system, as evidenced by neuroimaging data (66). Overall, eTNS appears effective in improving subjective sleep quality, but its impact on objective sleep measures remains to be validated in larger-scale, well-controlled trials. Future studies should consider insomnia subtypes (e.g., difficulty initiating sleep, impaired sleep maintenance, and early morning awakening) and baseline arousal levels in stratified analyses to enhance sensitivity and reproducibility.

In the field of disorders of consciousness, eTNS offers a novel approach for clinical intervention in these conditions. In 2018, case reports suggested that eTNS may improve consciousness levels in patients with unresponsive arousal syndrome following brain injury (118). In 2022, Wu et al. reported that trigeminal nerve electrical stimulation combined with rhythmic music enhanced brain oscillatory activity and improved consciousness levels in patients with impaired consciousness (119). But this combined protocol differs from standard frontal eTNS devices and should therefore be interpreted as preliminary evidence for trigeminal-based multimodal stimulation rather than direct evidence for routine clinical eTNS. In 2023, a randomized, double-blind, sham-controlled trial involving patients with prolonged disorders of consciousness further demonstrated that eTNS significantly improved clinical scores in severe cases. FDG-PET imaging further revealed increased glucose metabolism in consciousness-related brain networks, providing neuroimaging evidence supporting the arousal mechanism of eTNS (91). These findings suggest that eTNS may serve as a noninvasive intervention strategy to promote neurological recovery following severe brain injury. However, the current evidence remains limited by small sample sizes, substantial etiological heterogeneity, and short follow-up durations. Future studies should perform stratified analyses based on etiology, distinguishing among traumatic, hypoxic, and post-stroke causes of secondary disorders of consciousness.

## 6 Challenges in clinical practice

### 6.1 Safety and adverse events

Among patients with neuropsychiatric disorders, eTNS has demonstrated generally good tolerability overall. Multiple systematic reviews and clinical trials have indicated that adverse events associated with eTNS are typically mild, transient, and reversible, with no serious adverse events definitively attributed to the intervention (5, 7, 12, 14). The most commonly reported adverse effects include skin irritation and mild headache, while sleep-related symptoms have also been noted in some studies (6, 7). Most mild adverse reactions did not lead to treatment discontinuation and gradually subsided with continued use. Several studies have suggested that appropriate adjustment of stimulation parameters may improve overall tolerance (7, 120).

Skin irritation is among the most frequently reported adverse events, typically presenting as localized erythema, itching, stinging or prickling sensations, or mild pain (6, 7). The causative factors may include contact dermatitis and hypersensitivity reactions related to electrode materials. In parallel, general dosimetry studies on transcutaneous electrical stimulation have suggested that higher current intensities and current densities can increase skin discomfort, and individual skin sensitivity may further contribute to elevated risk; however, direct evidence specific to eTNS remains limited (6, 120, 121). Based on clinical experience, the use of hypoallergenic electrode patches, rotation of application sites, and maintenance of skin hygiene are often recommended to minimize the risk of local irritation or contact dermatitis; however, these preventive strategies have not been systematically validated (6, 120, 121). In rare cases, contact dermatitis may develop, in which case stimulation should be discontinued and appropriate local treatment initiated. If symptoms are severe, clinicians may consider using an alternative electrode type or reducing the frequency of stimulation; however, available evidence remains primarily empirical (120, 121).

Headache is another commonly reported adverse event associated with eTNS, typically mild in intensity, transient in duration, and seldom resulting in treatment discontinuation (6). In clinical practice, tolerance to mild headaches may be improved by reducing stimulation intensity, shortening stimulation duration, or applying individualized incremental parameters set near or slightly above the sensory threshold (120). If headaches persist, recur, or interfere with daily functioning, treatment should be temporarily discontinued, stimulation parameters reassessed, and other potential causes excluded.

Although eTNS has shown good efficacy in the treatment of a variety of neuropsychiatric disorders, its associated adverse events still warrant attention. When implementing eTNS treatment, clinicians should fully inform patients of possible adverse reactions and closely monitor patients’ responses during treatment, allowing timely adjustment of the treatment plan to ensure patient safety and optimize therapeutic outcomes. Additionally, caution should be exercised, or the therapy should be avoided, in patients with implanted cardiac pacemakers or defibrillators, deep brain stimulation systems, or other active implantable electronic devices, as well as in individuals with open wounds, active dermatitis, or severe skin lesions at the electrode placement site.

### 6.2 Methodological limitations of eTNS studies

In eTNS studies, the treatment inherently produces a mild tingling or vibrating sensation in the forehead, making it possible for participants to discern whether they have received genuine stimulation. This poses a risk of unblinding in single- or double-blind designs, thereby potentially compromising the quality assessment of the research evidence. Recent systematic reviews have highlighted the need for future randomized controlled trials with rigorous blinding procedures and adequate sample sizes to further validate the efficacy of eTNS and to provide more reliable dose–response evidence, while urging cautious interpretation of the currently positive findings (7).

First, a sham stimulation lacking any perceptible sensation or electrical output is used, while maintaining an identical device appearance and operational procedure to the active condition, as implemented in double-blind trials for ADHD (5). Second, by adjusting stimulation parameters such as frequency, intensity, or pulse width, sham stimuli can be engineered to replicate the sensory experience of genuine stimulation while lacking therapeutic efficacy. For example, in the TEAM trial involving patients with acute migraine, the active stimulation was delivered at 100 Hz, whereas the sham stimulation was set at 3 Hz. Both conditions used an identical pulse width of 250 μs and device appearance. Independent validation experiments confirmed that participants had difficulty distinguishing between active and sham stimulation (9). Third, low-intensity electrical stimulation can be used as a control, which helps maintain comparable sensory perception but may elicit partial therapeutic effects, thereby reducing between-group effect sizes. For instance, in a randomized controlled trial involving patients with DRE, the treatment group received 120 Hz stimulation, whereas the control group received low-frequency 2 Hz stimulation (106).

To enhance the reliability and reproducibility of research findings, sham stimulation protocols that closely mimic real stimuli in sensory experience, yet do not produce therapeutic effects, are recommended. Additionally, the efficacy of blinding methods should be evaluated during trials, for example by recording participants’ or evaluators’ guesses regarding group assignments and their accuracy rates, to assess whether blinding was successfully achieved (7, 9). Primary endpoints, planned sample size, and statistical analysis strategies should be clearly registered on a public platform prior to trial initiation, with complete reporting of stimulation parameter settings in the final publication. Binary or count-type endpoints should also be used whenever possible, and patient adherence carefully documented (9). If the control group receives low-dose stimulation, the sample size should be appropriately increased and sensitivity analysis conducted, taking into account its potential efficacy.

### 6.3 Disease-specific optimization of eTNS stimulation parameters

The efficacy of eTNS largely depends on the configuration of stimulation parameters, including frequency, pulse width, intensity, duty cycle, session duration, daily frequency, and total treatment course. Optimizing stimulation parameters requires balancing therapeutic efficacy with safety (15). Existing RCTs and systematic reviews have demonstrated that eTNS yields consistent therapeutic benefits in disorders such as ADHD, migraine, and DRE. However, the optimal parameter ranges differ among disease types; therefore, treatment protocols should be developed by stratifying patients based on specific disorders and subsequently refining stimulation parameters through individualized adjustments (5, 7, 13).

In the treatment of ADHD, the most common protocol entails home-based stimulation administered during nighttime sleep for approximately 8 hours. The stimulation parameters are typically configured with a frequency of 120 Hz, a pulse width of 250 μs, and a duty cycle of 30 s on and 30 s off, incorporating a 1 s gradual ramp-up and ramp-down phase. Clinical protocols typically recommend initiating stimulation at low intensities slightly above the sensory threshold, modifying only one major parameter (e.g., intensity or session duration) every 1 to 2 weeks. Improvements on symptom rating scales are generally observed within approximately 4 weeks, demonstrating favorable tolerability. This low-intensity, long-duration, and intermittent duty-cycle protocol minimizes skin discomfort and thereby supports stable implementation in home environments (5, 13).

For migraine, it is essential to differentiate between prophylactic and acute treatment approaches. Prophylactic treatment typically involves daily stimulation for 20–30 min over 8–12 consecutive weeks to assess changes in monthly migraine days and the 50% responder rate, whereas acute treatment is delivered for 1–2 h at the onset of an attack to evaluate pain freedom or relief of the most bothersome symptom at 2 hours. Stimulation parameters may vary slightly across devices; however, typical settings fall within a frequency range of 60–100 Hz and a pulse width of approximately 250 μs. In both study design and reporting, it is essential to explicitly define the treatment modality (acute or prophylactic) and ensure its alignment with the corresponding outcome measures. This practice helps prevent misinterpretation of findings arising from mismatched stimulation parameters and clinical outcomes (6, 9, 15, 16, 46).

In patients with DRE, RCTs commonly employ a stimulation protocol with a frequency of approximately 120 Hz, a pulse width of ≤250 μs, and a 30 s on/30 s off duty cycle, administered for 20–30 min daily. Treatment should begin at low intensity, with only one primary parameter adjusted every 1 to 2 weeks, while maintaining stable antiepileptic medication regimens to enable accurate evaluation of efficacy and safety. Follow-up assessments are primarily based on seizure diaries, with regular evaluations of the ≥50% responder rate and changes in median seizure frequency (47, 106, 110).

At present, foundational research on eTNS stimulation parameters remains limited, and no standardized guidelines for parameter configuration have been established. Optimal stimulation parameters likely differ among disorders; therefore, treatment protocols should incorporate both disease-specific characteristics and interindividual variability to enable personalized therapeutic planning. Further large-scale, multicenter clinical trials are warranted to identify optimal stimulation parameters across conditions, thereby maximizing therapeutic efficacy while minimizing adverse events.

## 7 Discussion

This review offers a comprehensive synthesis of the anatomical foundations, multi-level mechanisms, and clinical evidence regarding eTNS in various neuropsychiatric disorders. Existing studies generally suggest that eTNS is associated with clinically meaningful improvement and favorable tolerability in conditions such as ADHD, migraine, and epilepsy (5, 9, 13, 16, 46, 47, 106). The mechanisms of action encompass multi-level and multi-pathway processes, including neurotransmitter modulation, alterations in synaptic plasticity, neurovascular–immune interactions, and functional reorganization within large-scale brain networks such as the DMN, CEN, and SN networks. The relative contribution of these mechanisms may vary among different disorders and symptom profiles. This not only provides a theoretical basis for the transdiagnostic application of eTNS but also suggests that future research should further elucidate the structured integration of its multi-level mechanisms.

Overall, the primary strengths of current clinical studies reside in the consistent demonstration of favorable safety profiles and patient compliance (5, 7, 12, 14). However, several notable limitations remain. Most studies are characterized by small sample sizes and short follow-up durations, which restrict the evaluation of long-term efficacy and safety. Moreover, stimulation-induced facial sensations may compromise blinding procedures and increase susceptibility to placebo effects (7). In addition, marked variability in stimulation parameters—including frequency, pulse width, intensity, duty cycle, daily dose, and treatment duration—persists across studies, and the absence of standardized parameter protocols hinders comparability of findings and impedes clarification of the dose–response relationship. Future studies should routinely report blinding efficacy (e.g., group-guess accuracy), treatment adherence, and participant dropout rates, and employ sensitivity and subgroup analyses to minimize potential bias.

At the mechanistic level, existing evidence primarily centers on four complementary pathways: (1) Neurotransmitter regulation: eTNS modulates arousal, emotional, and attentional networks through activation of the LC–NE and DRN–5-HT systems (5, 7, 12, 16, 42, 46, 47); (2) Neural plasticity: Repeated stimulation induces LTP-like or LTD-like responses, which may account for its sustained therapeutic effects (60, 69–71); (3) Neurovascular–immune mechanisms: This pathway has attracted considerable attention, particularly in migraine. eTNS may enhance neurovascular function by modulating neuropeptide release and inflammatory activity (12, 75); and (4) Network integration: eTNS may exert transdiagnostic effects through bidirectional coordination between ascending inputs from the brainstem and thalamus and descending regulation from the PFC and ACC (38, 104, 105). Nevertheless, most mechanistic studies remain confined to a single level of analysis, lacking a continuous validation framework that links neural activity, molecular markers, network dynamics, and clinical outcomes. Furthermore, integrated analyses of eTNS mechanisms across different disorders and symptom domains remain scarce, hindering the identification of shared neural circuits and distinct regulatory pathways that underlie its transdiagnostic therapeutic effects.

Based on existing evidence and clinical demands, future research should aim to establish a “stratified–closed-loop–integrated” research framework and translational pathway. Prior to intervention, patient stratification should be conducted using baseline neurophysiological and neuroimaging biomarkers—such as quantitative EEG, pupillary responses, heart-rate variability, and resting-state functional connectivity—to identify high-responder subgroups. During intervention, real-time physiological feedback can be utilized to develop closed-loop stimulation systems that enable adaptive parameter optimization. In parallel, molecular, neuroimaging, and clinical outcome metrics should be integrated to construct a “stimulation–molecule–network–behavior” systems model, thereby elucidating multilevel causal mechanisms and informing personalized therapeutic strategies.

In summary, eTNS has demonstrated promising efficacy, a favorable safety profile, and good treatment adherence across several neuropsychiatric disorders, including ADHD, migraine, and epilepsy. In clinical practice, patients should be stratified by disorder type, with minor individualized adjustments to stimulation parameters—including frequency, pulse width, intensity, duty cycle, and daily dose—to optimize real-world efficacy and tolerability. Integration of physiological and neurofunctional biomarkers—such as pupillary responses, heart-rate variability, and EEG or functional connectivity metrics—may further enhance response prediction and support closed-loop modulation strategies. This review employed a structured search approach to summarize the existing clinical and mechanistic literature on eTNS and conducted a concept-driven narrative synthesis that links anatomical foundations, mechanisms of action, stimulation parameters, and clinical outcomes. We did not perform a formal risk-of-bias assessment or evidence grading; thus, our conclusions focus on the breadth and consistency of existing evidence and identify priorities for future research, rather than providing quantitative effect size estimates. Future validation should be conducted through multicenter randomized controlled trials and studies on parameter standardization, and research design and reporting should follow current methodological guidelines.
