# Supplementary material for: External trigeminal nerve stimulation for neuropsychiatric disorders: mechanisms, efficacy, and future directions
Source: Front Neurol. 2026 Jan 23;16:1737506. doi: 10.3389/fneur.2025.1737506 (PMC12875929; doi:10.3389/fneur.2025.1737506)
Supplement: Supplementary file 1 [file Data_Sheet_1.PDF]

Supplementary file 1: Search strategy

Electronic Databases: PubMed, Embase, and Web of Science.

PubMed Search Strategy:

#1 "Transcutaneous Electric Nerve Stimulation"[MeSH Terms]

#2 "Trigeminal Nerve"[MeSH Terms]

#3 #1 OR #2

#4 "external trigeminal nerve stimulation"[Title/Abstract] OR "supraorbital nerve stimulation"[Title/Abstract] OR "trigeminal nerve stimulation"[Title/Abstract] OR "supraorbital transcutaneous nerve stimulation"[Title/Abstract] OR "transcutaneous supraorbital nerve stimulation"[Title/Abstract] OR "Cefaly"[Title/Abstract] OR "eTNS"[Title/Abstract] OR "e-TNS"[Title/Abstract] OR "t-SNS"[Title/Abstract]

#5 #3 OR #4

Embase Search Strategy:

#1 'trigeminal nerve'/exp

#2 'transcutaneous electrical nerve stimulation'/exp

#3 #1 AND #2

#4 'external trigeminal nerve stimulation':ab,ti OR 'supraorbital nerve stimulation':ab,ti OR 'trigeminal nerve stimulation':ab,ti OR 'supraorbital transcutaneous nerve stimulation':ab,ti OR 'transcutaneous supraorbital nerve stimulation':ab,ti OR 'Cefaly':ab,ti OR 'eTNS':ab,ti OR 'e-TNS':ab,ti OR 't-SNS':ab,ti

#5 #3 OR #4

Web of Science Search Strategy:

#1 TS=(Trigeminal Nerve)

#2 TS=(Transcutaneous Electric Nerve Stimulation)

#3 #1 AND #2

#4 TS=(external trigeminal nerve stimulation OR supraorbital nerve stimulation OR

trigeminal nerve stimulation OR supraorbital transcutaneous nerve stimulation OR transcutaneous supraorbital nerve stimulation OR Cefaly OR eTNS OR e-TNS OR t-SNS)
#5 #3 OR #4
